# Changes in expression of insulin signaling pathway genes by dietary fat source in growing-finishing pigs

**DOI:** 10.1186/2055-0391-56-12

**Published:** 2014-08-01

**Authors:** Seung-Chang Kim, Hong-Chul Jang, Sung-Dae Lee, Hyun-Jung Jung, Jun-Cheol Park, Seung-Hwan Lee, Tae-Hun Kim, Bong-Hwan Choi

**Affiliations:** Animal Genomics & Bioinformatics Division, National Institute of Animal Science, Rural Development Administration, Chuksan-gil 77, Kwonsun-gu, Suwon, Korea; Swine Science Division, National Institute of Animal Science, Rural Development Administration, Cheon-an, Chungnam, 330-801 Korea

**Keywords:** Dietary fat, Gene expression, Growing-finishing pig, Insulin signaling pathway

## Abstract

This study investigated changes in gene expression by dietary fat source, *i.e.*, beef tallow, soybean oil, olive oil, and coconut oil (each 3% in feed), in both male and female growing-finishing pigs. Real-time PCR was conducted on seven genes (insulin receptor; INSR, insulin receptor substrate; IRS, phosphatidylinositol (3,4,5)-triphosphate; PIP3, 3-phosphoinositide-dependent protein kinase-1; PDK1, protein kinase B; Akt, forkhead box protein O1; FOXO1 and cGMP-inhibited 3’, 5’-cyclic phosphodiesterase; PDE3) located upstream of the insulin signaling pathway in the *longissimus dorsi* muscle (LM) of pigs. The INSR, IRS, PIP3, and PDE3 genes showed significantly differential expression in barrow pigs. Expression of the PIP3 and FOXO1 genes was significantly different among the four dietary groups in gilt pigs. In particular, the PIP3 gene showed the opposite expression pattern between barrow and gilt pigs. These results show that dietary fat source affected patterns of gene expression according to animal gender. Further, the results indicate that the type of dietary fat affects insulin signaling-related gene expression in the LM of pigs. These results can be applied to livestock production by promoting the use of discriminatory feed supplies.

## Background

Fat supplementation with high energy value is important for growing-finishing pigs. Addition of dietary fat has been shown to improve feed efficiency during the post-weaning period [1–5]. Dietary fat type affects fatty acid composition in the LM [6]. Intramuscular fat (IMF) deposition and back fat thickness (BF) are the most important candidate traits for understanding the interactions between nutrition and gene expression in pigs [7].

The insulin signaling pathway has a well established relationship with fat metabolism. Therefore, genes related to the insulin signaling pathway have long been the subject of major research. Insulin is the major hormone for fatty acid synthesis, glycolysis, and glycogenesis, and it suppresses β-oxidation, gluconeogenesis, glycogenolysis, and apoptosis by controlling critical energy functions such as glucose and lipid metabolism [8–10]. Insulin activates insulin receptor (IR), which is a tyrosine kinase that phosphorylates and recruits different substrate adaptors such as the IRS family of proteins. Phosphorylated IRS then displays binding sites for numerous signaling partners. Among them, PI3K plays a major role in insulin function, mainly via activation of the Akt/PKB pathway. Activated Akt induces anti-lipolysis through activation of PDE3 as well as regulates gluconeogenesis and glycogenolysis through inhibition of forkhead box protein O1 (FOXO1). Activation of PDE3 decreases the concentration of cAMP, which in turn reduces protein kinase A (PKA) activity [11]. PKA is responsible for activation of lipase, which induces lipolysis as well as other physiological pathways [12]. Inhibition of FOXO1 decreases transcription of glucose 6-phosphates, which consequently reduces rates of gluconeogenesis and glycogenolysis [13]. Protein phosphorylation is controlled by the opposing and coordinated activities of protein kinases and phosphatases catalyzing protein phosphorylation and dephosphorylation, respectively [14]. This reversible phosphorylation of proteins is a major mechanism responsible for the regulation of cellular functions, including metabolism, signal transduction, cell division, and memory [15].

The Akt/PI3K signaling pathway is crucial to cell growth and survival. As such, current research has attempted to develop anti-cancer drugs based on the Akt/PI3K signaling pathway. Further, many studies have focused on mechanisms related to glucose uptake via Glut4 as well as protein synthesis via mTOR in the insulin signaling pathway. However, little is known about other pathway mechanisms. In this study, we investigated genes located upstream of the insulin signaling pathway related to glycolysis and anti-lipolysis in growing-finishing pigs.

## Methods

### Animals and diets

A total of 72 crossbred pigs (Landrace × Large White × Duroc) consisting of 36 gilt and barrow pigs each were used. The animals had an average body weight of 71 ± 1 kg, were about 130 days of age, and were divided according to gender. The pigs were randomly allocated into 24 pens (320 × 150 cm with solid concrete flooring) in a confined pig house, with three pigs per pen and six replicate pens per treatment. Treatment groups consisted of the same numbers of gilts and barrows. Each pen was equipped with a nipple water bottle and a stainless steel feeder, and pigs were given free access to feed and water throughout. Animals received care in accordance with the standard guideline for the Care and Use of Laboratory Animals provided by the National Institute of Animal Science Animal Care Committee, and the experiment was conducted with approval from the animal ethics committee and Operation rule of animal experiment ethics in the National Institute of Animal Science (approval number: 2009–076).

The ingredients and chemical compositions of the growing and finishing diets used in this experiment are shown in Table 1. All other nutrient requirements met or exceeded NRC recommendations for growing and finishing pigs (NRC, 1998). Dietary fat sources used in the present study were beef tallow, coconut oil, olive oil, and soybean oil, which were added to feed at a concentration of 3.0%. For this, fat sources were melted at approximately 50°C, after which they were diluted to approximately 10%. The 10% fat diets were then formulated to 3.0% fat diets. Growing diet was administered to crossbred pigs for an experimental period of 14 ± 3 days, whereas finishing diet was administered to crossbred pigs for an experimental period of 28 ± 3 days.

**Table 1:** **Composition of experiment diets, as-fed basis**

Items	Growing	Finishing
Ingredients, %		
Corn grain	62.38	57.64
Soybean meal	22.00	14.00
Wheat	10.00	11.00
Wheat bran	0.00	12.00
Fat source^1)^	3.00	3.00
L-lysine	0.06	0.06
Limestone	0.65	1.10
Tricalcium phosphate	1.11	0.30
Sodium chloride	0.30	0.30
Vitamin + mineral premix^2)^	0.40	0.40
Antibiotics	0.10	0.00
Chemical composition^3)^		
DE, kcal/kg	3,500	3,400
Crude protein, %	15.44	13.42
Crude fat, %	5.50	5.67
Crude fiber, %	3.45	3.94
Lysine,%	0.82	0.66
Methionine + Cystine, %	0.52	0.47
Calcium, %	0.69	0.60
Phosphorus, %	0.54	0.47

^1)^Fat source : Beef tallow, soybean oil, olive oil, coconut oil.

^2)^Vitamin and mineral contents per kilogram of diet provided by premix: Vitamin A, 2,000,000 IU; Vitamin D_3_, 400,000 IU; Vitamin E, 2,500 IU; Vitamin K_3_, 100 mg; Vitamin B_1_, 100 mg; Vitamin B_2_, 300 mg; Vitamin B_12_, 1,200mcg; Niacin, 2,000 mg; d-Pantothenicalcium, 1,000 mg; Folic acid, 200 mg; Biotin, 20 mg; Choline chloride, 25,000 mg; Mn, 12,000 mg; Zn, 15,000 mg; Fe, 4,000 mg; Cu, 500 mg; I, 250 mg; Co, 100 mg; Mg, 2,000 mg; B.H.T., 5,00 mg.

^3)^Chemical composition was calculated from ingredient proportion.

### Slaughtering and sampling

Pigs with a live weight of 102 ± 3 kg were transported to a standard abattoir near the experimental station. The pigs were then slaughtered at 12 h after feed restriction. Briefly, pigs were stunned electrically (300 V for 3 s) with a pair of stunning tongs, shackled by the right leg, and exsanguinated while hanging. The carcasses were placed in a dehairer at 62°C for 5 min, and remaining hair was removed using a knife and flame. The carcasses were eviscerated and split before being placed in a chiller set at 4°C for 12 h. Immediately, 24 LM samples were taken from animals in the four dietary groups, frozen in liquid nitrogen, and stored at -80°C until preparation of total RNA.

### RNA isolation and cDNA synthesis

The tissue was powdered with liquid nitrogen, and total RNA was extracted from 10 mg of muscle tissue using 1 mL of TRIzol® reagent (Invitrogen, Inc., USA). RNA quality was confirmed by examining 28S and 18S rRNA bands on 1.5% agarose gels stained with ethidium bromide. Total RNA was purified from all samples using an RNeasy MinElute cleanup kit (Qiagen, USA).

Complementary DNA (cDNA) synthesis was performed by reverse transcription using SuperScript™ II reverse transcriptase (Invitrogen, USA) as follows. Aliquots (4 μL) of total RNA were preincubated with 50 ng (1 μL) of random primer mix (Promega, USA) and 2.5 mM (1 μL) dNTP mix at 65°C for 5 min. The tubes were placed on ice, after which 4 μL of 5× first-stand buffer (250 mM Tris–HCl, pH 8.3, 375 mM KCl, 15 mM MgCl_2_), 2 μL of 0.1 M DTT, and 40 units (0.5 μL) of RNase inhibitor (Promega, USA) were added, followed by incubation at 42°C for 2 min. After addition of 200 units (1 μL) of SuperScript™ II reverse transcriptase (Invitrogen, USA), incubation was continued at 42°C for 50 min. Reverse transcriptase activity was terminated by incubation at 70°C for 15 min. The resulting cDNA was stored at −20°C until used in quantitative real-time PCR (qRT-PCR).

### Quantitative real-time polymerase chain reaction (qRT-PCR) analysis

To validate the seven differentially expressed genes (DEGs) related to insulin signaling based on the KEGG database in the LM of pigs, we performed qRT-PCR using Power SYBR Green PCR Master Mix (Applied Biosystems, USA) and the ABI 7500 Real-Time PCR system (Applied Biosystems USA). All primer sets were designed using the Primer3 program (http://bioinfo.ut.ee/primer3-0.4.0/) to amplify products ranging from 100 to 200 base pairs (Table 2). The β-actin gene (GenBank Acc. No. AY550069) was used as an internal control. qRT-PCR was performed in a total volume of 20 μL containing 2 μL of cDNA (0.1 μg/μL), 10 μL of 2× SYBR® Green PCR Master Mix (Applied Biosystems, USA), and 1 μL each of 10 pM forward and reverse primers. The amplification reaction was initiated by incubation for 2 min at 50°C, followed by 40 cycles of 95°C for 10 min, 95°C for 10 s, and 60°C for 1 min. After 36 cycles, a final extension step was performed at 72°C for 1 min. qRT-PCR for each gene was repeated three times. Following amplification, melting curve analysis was performed to verify the specificity of the reactions. The endpoint used in real-time RT-PCR quantification (Ct) was defined as the PCR threshold cycle number. The ΔCt value was determined by subtracting the β-actin Ct value for each sample from the target Ct value. Finally, we transformed the expression level to the 2^-ΔCt^ value for further analysis.

**Table 2:** **List of insulin signaling pathway primers for qPCR**

Gene Symbol	Primer sequences Forward / Reverse	Product Size (bp)
INSR	F:5′-TTCACTGGCAATCGCATTGAGCTG-3′	137 bp
	R:5′-TCATGGGTCACAGGGCCAATGATA-3′	
IRS	F:5′-AGGAAGTTTGGCAGGTGATCCTGA-3′	200 bp
	R:5′-ACGGCCCACTTCGATGAAGAAGAA-3′	
PIP3	F:5′-CTTTGCAGAGCTTGACCCAGAT-3′	100 bp
	R:5′-GAGCTTGTGGGCTTGCCTTCATTT-3′	
PDK1	F:5′-GGAAACCCTTGGCACCAGTTTGTA-3′	183 bp
	R:5′-TCGGAGTTCTTGTGACCACGGAAT-3′	
Akt	F:5′-AGAAGCTCTTCGAGCTCATCCTCA-3′	148 bp
	R:5′-TGCATGATCTCCTTGGCATCCTCA-3′	
FOXO1	F:5′-TCCCACACAGTGTCAAGACAACGA-3′	118 bp
	R:5′-ACTGCTTCTCTCAGTTCCTGCTGT-3′	
PDE3	F:5′-CCTGCAGAACCACAAGATGTGGAA-3′	190 bp
	R:5′-TCACTGGTTTGGCTTTGGTGTTGG-3′	

INSR, insulin receptor; IRS, insulin receptor substrate; PIP3, phosphatidylinositol 3-kinase; PDK1, 3-phosphoinositide-dependent protein kinase-1; Akt, protein kinase B; FOXO1, forkhead box protein O1; PDE3, cGMP-inhibited 3’,5’-cyclic phosphodiesterase.

### Statistical analysis

To identify DEGs among the dietary fat groups, statistical analysis was performed by analysis of variance (ANOVA) using the MIXED procedure with the R statistical package ( http://www.R-project.org) for animals nested within age as the random effect. We also examined the least square means (LSM) to test the significance of differences among the groups using Duncan’s multiple range test. The following statistical model was used to estimate the effects of dietary fat type on individual gene expression:


Where *Y*_*ij*_ is the target gene intensity (2^-ΔCt^), *μ* is the overall mean, *FED*_*i*_ is the fixed effect of the *i*th dietary type, and *DAY*_*j*_ is animals nested within age as a random effect.

## Results and discussion

Animal fat sources such as beef tallow have low digestibility, which can be improved by mixing animal fat with various vegetable oils to increase meat quality via elevation of lipase activity [16–19]. Therefore, gene expression in the LM can be manipulated according to dietary nutrients.

DNA microarray analysis has previously revealed that dietary fat type influences LM gene expression profiles. In particular, expression of insulin signaling pathway-related genes has been shown to be significantly enriched in differential gene expression sets [6]. These changes also suggest significant changes in other insulin signaling pathway genes. Thus, we compared differential gene expression in the LM of three barrows and gilts for each dietary fat type. Various genes linked to insulin signaling pathway genes as well as differentially expressed genes identified through microarray analysis were confirmed by RT-PCR.

The insulin signaling pathway involves a number of genes that control glucose storage and uptake, protein synthesis, and regulation of lipid synthesis in pigs. Insulin inhibits lipid metabolism by activating a cAMP-specific phosphodiesterase in adipocytes, thereby reducing cellular cAMP concentrations [20]. As the insulin signaling pathway is closely related to fat metabolism [21], the seven genes were subjected to qRT-PCR to determine whether or not dietary fat type influences their expression in pigs. The seven genes were insulin receptor (INSR), insulin receptor substrate (IRS), phosphatidylinositol (3,4,5)-triphosphate (PIP3), 3-phosphoinositide-dependent protein kinase-1 (PDK1), protein kinase B (Akt), forkhead box protein O1 (FOXO1), and cGMP-inhibited 3’, 5’-cyclic phosphodiesterase (PDE3).

**Figure 1:**
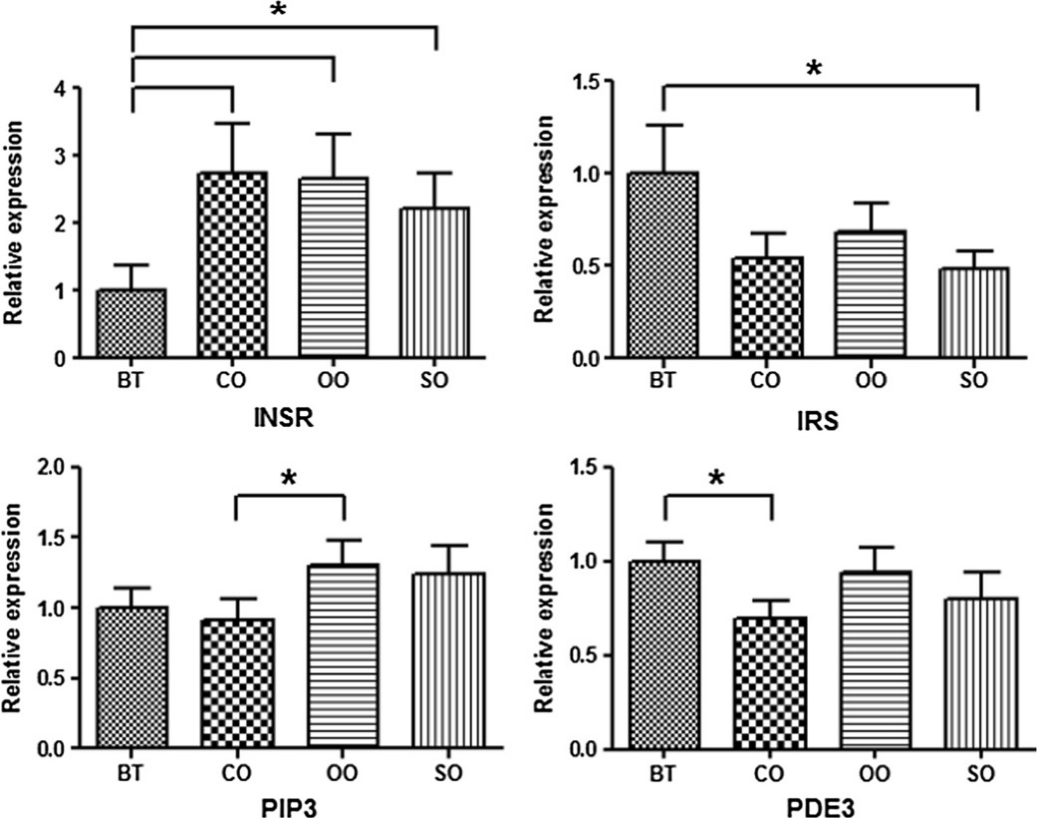
**Expression patterns of differentially expressed genes in four dietary oil groups in barrows, as determined by qRT-PCR.** Dietary fat sources were BT (beef tallow), CO (coconut oil), OO (olive oil), and SO (soybean oil), which were added at concentrations of 3.0% in feed. Experiments were performed using the LM from three barrows, and data are expressed as mean ± SD. Asterisks show statistically significant values (*P < 0.05).

Expression levels of the seven DEGs were measured using the ΔCt method, and the results are shown in Tables 3 and 4. RT-PCR analysis determined the expression profiles of the insulin signaling pathway genes in growing-finishing pigs. In particular, the INSR, IRS, PIP3, and PDE3 genes showed significantly differential expression according to dietary oil composition in barrow pigs (Figure 1). INSR plays a key role in the regulation of glucose homeostasis, a functional process that may result in a range of clinical manifestations, including diabetes and cancer, under degenerate conditions [22]. IRS plays a key role in transmitting signals from insulin and insulin-like growth factor-1 (IGF-1) receptors to the intracellular Akt/PI3K and Erk/MAPK pathways [23]. PIP3 functions to activate downstream signaling components such as protein kinase Akt, which activates downstream anabolic signaling pathways required for cell growth and survival [24]. Especially, PDE3 undergoes phosphorylation and short-term activation in response to insulin as well as agents that increase cAMP in adipocytes, hepatocytes, and platelets [11].

**Table 3:** **ANOVA table for each gene associated with feeding groups in barrows**

Gene	Source	Df	Sum Sq	Mean Sq	F value	Pr(>F)
INSR	Feed	3	2.084	0.695	4.014	<0.05*
	Residuals	20	3.461	0.173		
IRS	Feed	3	1.450	0.483	3.868	<0.05*
	Residuals	20	2.499	0.125		
PIP3	Feed	3	0.422	0.141	4.370	<0.05*
	Residuals	20	0.643	0.032		
PDK1	Feed	3	0.100	0.033	0.396	0.758
	Residuals	20	1.683	0.084		
Akt	Feed	3	0.209	0.070	1.021	0.405
	Residuals	20	1.365	0.068		
FOXO1	Feed	3	1.527	0.509	1.880	0.166
	Residuals	20	5.416	0.271		
PDE3	Feed	3	0.927	0.309	5.213	<0.01**
	Residuals	20	1.186	0.059		

INSR, insulin receptor; IRS, insulin receptor substrate; PIP3, phosphatidylinositol (3,4,5)-triphosphate; PDK1, 3-phosphoinositide-dependent protein kinase-1; Akt, protein kinase B; FOXO1, forkhead box protein O1; PDE3, cGMP-inhibited 3’,5’-cyclic phosphodiesterase. *, ** Significant differences (P < 0.05 and 0.01) among the feeding groups determined using the mixed ANOVA module. DF = Degrees of freedom, Sum Sq = Sum of square, Mean Sq = Mean of square.

**Table 4:** **ANOVA table for each gene associated with feeding groups in gilts**

Gene	Source	Df	Sum Sq	Mean Sq	F value	Pr(>F)
INSR	Feed	3	0.457	0.153	0.923	0.448
	Residuals	20	3.304	0.165		
IRS	Feed	3	0.197	0.066	0.249	0.861
	Residuals	20	5.268	0.263		
PIP3	Feed	3	0.970	0.323	4.644	<0.05*
	Residuals	20	1.393	0.070		
PDK1	Feed	3	0.386	0.129	1.258	0.316
	Residuals	20	2.043	0.102		
Akt	Feed	3	0.308	0.103	1.380	0.278
	Residuals	20	1.486	0.074		
FOXO1	Feed	3	2.712	0.904	7.830	<0.01**
	Residuals	20	2.309	0.115		
PDE3	Feed	3	0.300	0.100	0.530	0.667
	Residuals	20	3.770	0.189		

INSR, insulin receptor; IRS, insulin receptor substrate; PIP3, phosphatidylinositol (3,4,5)-triphosphate; PDK1, 3-phosphoinositide-dependent protein kinase-1; Akt, protein kinase B; FOXO1, forkhead box protein O1; PDE3, cGMP-inhibited 3’,5’-cyclic phosphodiesterase. *, ** Significant differences (P < 0.05 and 0.01) among the feeding groups determined using the mixed ANOVA module. DF = Degrees of freedom, Sum Sq = Sum of square, Mean Sq = Mean of square.

The INSR and IRS genes showed opposite gene expression patterns in barrows. Beef tallow was the only dietary fat source associated with low INSR expression as well as increased IRS expression. The PIP3 and PDE3 genes showed similar reduced expression levels in pigs treated with coconut oil. In particular, INSR gene expression was up-regulated 2-fold in pigs treated with other oils compared to beef tallow.

**Figure 2:**
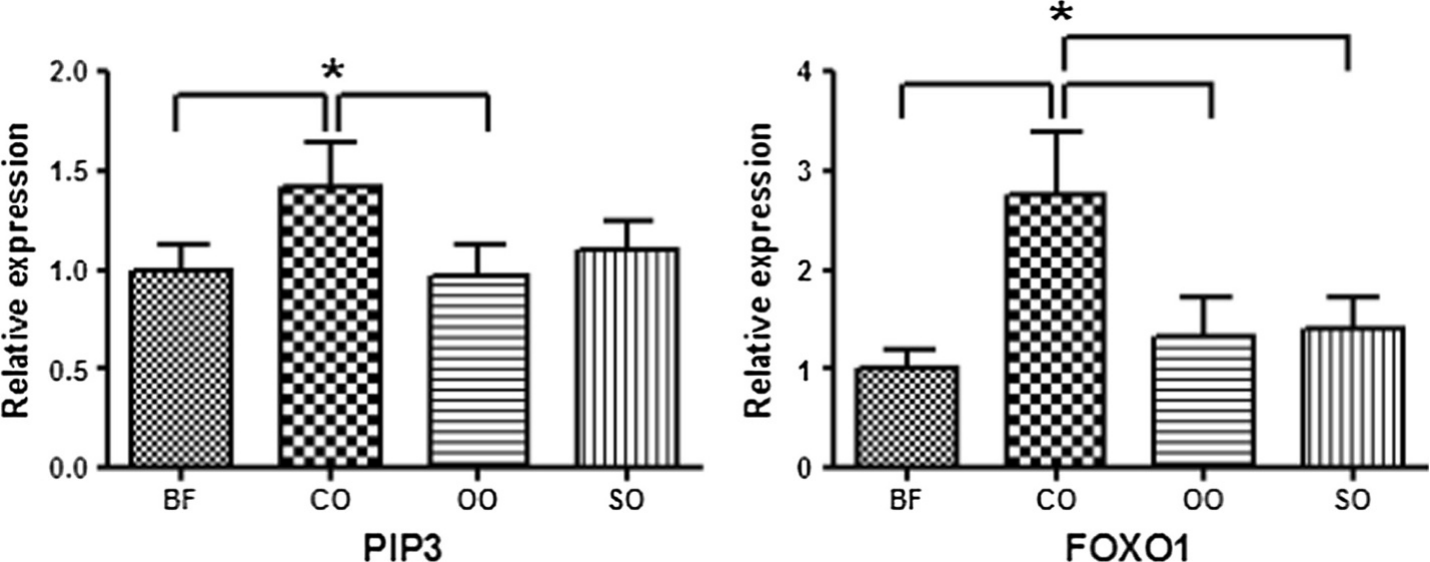
**Expression patterns of differentially expressed genes in four dietary oil groups in gilts, as determined by qRT-PCR.** Dietary fat sources were BT (beef tallow), CO (coconut oil), OO (olive oil), and SO (soybean oil), which were added to concentrations of 3.0% in feed. Experiments were performed using the LM from three gilts, and data are expressed as mean ± SD. Asterisks show statistically significant values (*P < 0.05).

The PIP3 and FOXO1 genes showed significantly different expression levels among the four dietary oil groups in gilts (Figure 2). Specifically, the PIP3 and FOXO1 genes showed relatively high expression in gilts with coconut oil compared to other oils. Gene expression of FOXO1, which negatively regulates adipogenesis, was two times higher in gilts [25]. FOXO1 belongs to the forkhead family of transcription factors that increases transcription of glucose-6-phosphatase, resulting in elevated rates of gluconeogenesis and glycogenolysis [26]. Compared with other oils, activation of FOXO1 in pigs treated with coconut oil had a negative effect on intramuscular fat accumulation.

Barrows and gilts showed opposite patterns of PIP3 gene expression. These results show that dietary fat type affected patterns of gene expression according to animal gender. It is known that expression levels of genes are linked to gender differences and variations in fatty acids [27, 28]. These results can be applied to livestock production by promoting the use of discriminatory feed supplies.

## Conclusion

DNA microarray analysis has previously revealed that dietary fat type alters LM gene expression profiles. These changes also suggest significant changes in other insulin signaling pathway genes. Thus, we compared differential gene expression in the LM of three barrows and gilts for each dietary fat type. Various genes linked to insulin signaling pathway genes as well as differentially expressed genes identified through microarray analysis were confirmed by RT-PCR. Results show that seven genes (INSR, IRS, PIP3, PDK1, Akt, PDE3, and FOXO1) were located upstream of the insulin signaling pathway. In barrows, the INSR, IRS, PIP3, and PDE3 genes showed significantly differential expression according to dietary oil type. The PIP3 and FOXO1 genes showed significant differences in gene expression among the four dietary oil groups in gilts. In particular, barrow and gilt pigs showed opposite patterns of PIP3 expression. Therefore, dietary fat type affected patterns of gene expression by gender difference. These results can be applied to livestock production by promoting the use of discriminatory feed supplies.
